# Supplementary material for: Structural insights into Frizzled3 through nanobody modulators
Source: Nat Commun. 2024 Aug 22;15:7228. doi: 10.1038/s41467-024-51451-1 (PMC11341715; doi:10.1038/s41467-024-51451-1)
Supplement: Supplementary file 1 — Supplementary Information [file 41467_2024_51451_MOESM1_ESM.pdf]

```
                                             CDR-1                        CDR-2
Nb-1  QVQLVESGGG LVQAGGSLRL SCAASGNIFS INAMGWYRQA AGKQRELVAA ITSGGSTNYA
Nb-3  QVQLVESGGG LVQAGGSLRL SCAASGSIFS INAMGWYRQA PGKQRELVAA ITSGGSTNYA
Nb-4  QVQLVESGGG LVQAGGSLRL SCAASGNIFS INDMGWYRQA PGKQRELVAA ITSGGITNYA
Nb-5  QVQLVESGGG LVQAGGSLRL SCAASGSIFS INAMGWYRQA PGKQRELVAT ITDGGSTNYA
Nb-6  QVQLVESGGG LVQAGGSLRL SCAASGSIFS VNAMGWYRQA PGKQRELVAV ITDGGSTNYG
Nb-8  QVQLVESGGG LVQAGGSLRL SCAASGSISS INAMGWYRQA PGKQRELVAA ITSGGSTNYA
Nb-9  QVQLVESGGG LVQAGGSLRL SCTASGRIFN LDVMGWYRQA PGKRRELVAD ITSGGKINYA

                                                      CDR-3
Nb-1  DSVKGRFTIS RDNAKNTVYL QMNSLKPEDT AVYYCNA--- DEWALGEHWG QGTQVTVSS
Nb-3  DSVKGRFTIS RDNAKNTVYL QMNSLKPEDT AVYVCNA--- DEWALGDHWG QGTQVTVSS
Nb-4  DSVKGRFTIS RDNAKNTVYL QMNSLKPEDT AVYYCNA--- EEWALGEYWG QGTQVTVSS
Nb-5  DSVKGRFTIS RDNAKNTVYL QMNSLKPEDT AVYYCNA--- DEWAVGEYWG QGTQVTVSS
Nb-6  DSVKGRFTIS RDNAKNTVYL QMRSLKSEDT AVYYCYA--- DEWAVGEYWG QGTQVTVSS
Nb-8  DSVKGRFTIS RDNAKNTVYL QMNSLKPGDT AVYYCNLLYY IDYVEYDYWG QGTQVTVSS
Nb-9  DSVKGRFTIS RDNTKDTVYL QMNSLKPEDT AVYYCNA--E VEWLDMDYWG KGTQVTVSS
```

Supplementary Fig. 1: Amino acid sequences of anti-FZD3 nanobodies.

Nanobodies raised against the full length FZD3 (including both the CRD and the transmembrane domain) from llama. Conserved amino acids are shown in black, partly conserved in blue and non-conserved in red. CDRs are indicated on the top of the sequences. Nb8 residues responsible for interactions with FZD3 CRD are highlighted in green. Nb9 residues responsible for interactions with the TM domain facing the cytoplasm are highlighted in yellow.

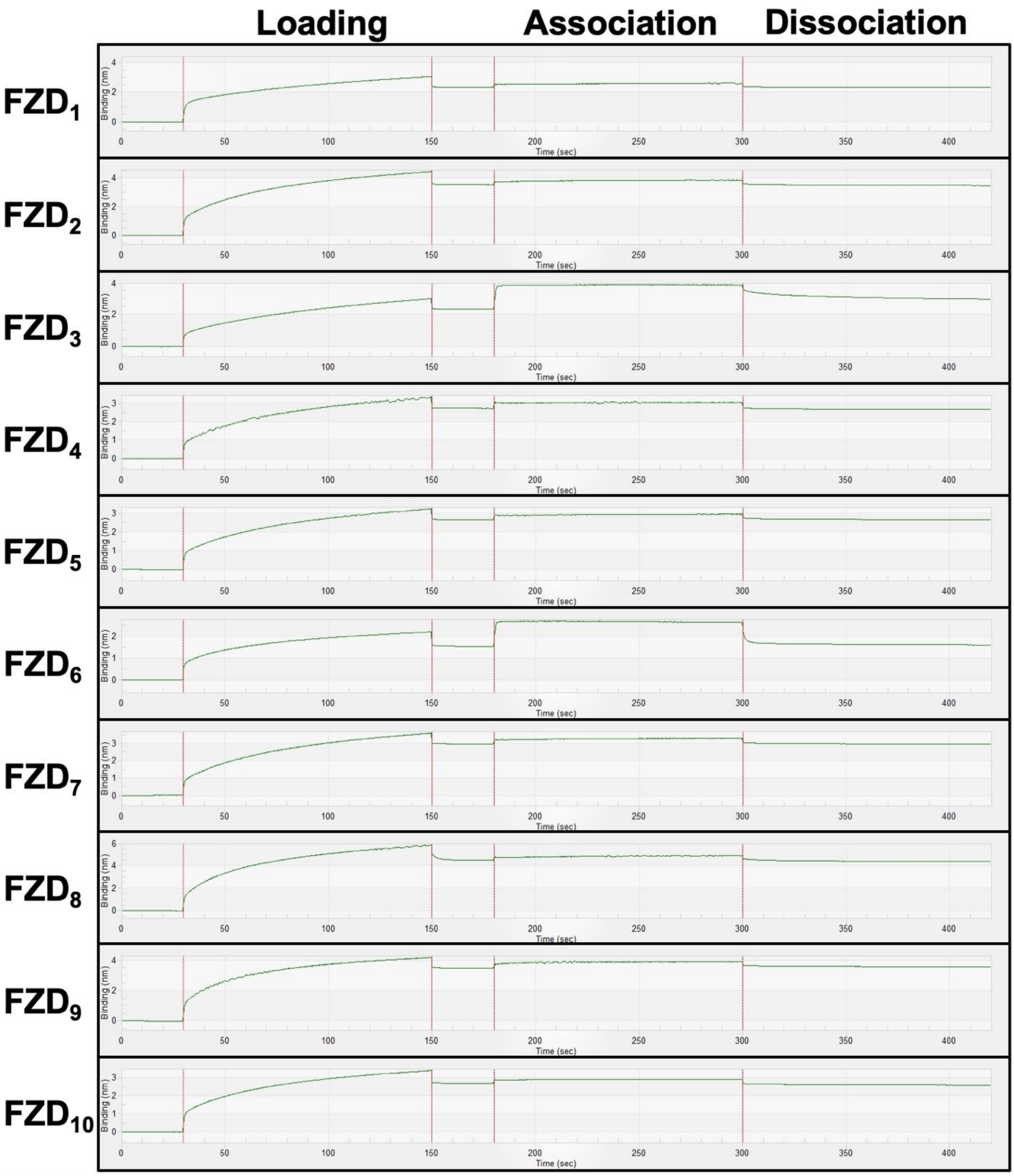

Supplementary Fig. 2: BLI Nb8 binding data for all 10 human FZD CRDs.

Biotinylated CRDs of FZDs 1-10 were separately loaded onto streptavidin-coated sensor tips and then dipped into Nb8 (5 µM) to measure binding responses with a BLItz System (ForteBio). The boundaries for the 10 human FZD CRD sequences were: FZD1 113-251; FZD2 36-158; FZD3 26-143; FZD4 42-179; FZD5 31-176; FZD6 22-139; FZD7 33-173; FZD8 28-153; FZD9 36-162; FZD10 31-157. Note that Nb8 associated only with FZD3 and FZD6.

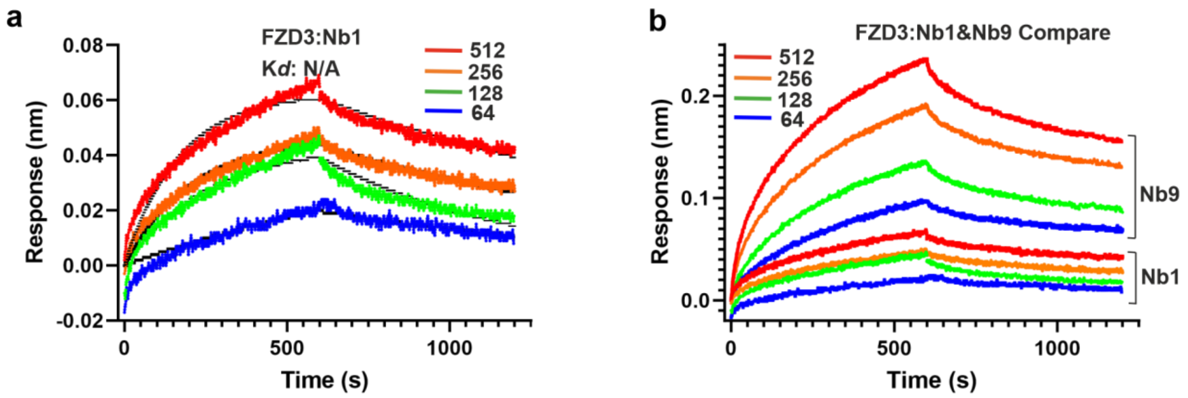

Supplementary Fig. 3: BLI based binding data for FZD3-Nb1 and FZD3-Nb9 interactions.

a Biotinylated FZD3 was loaded onto streptavidin coated BLI sensor tips and then exposed to different concentrations of Nb1. Binding responses are shown as coloured lines reflecting different nanobody concentrations (nM). Fitted curves are shown as black lines. In case of FZD3-Nb1, the overall binding responses were too small to suggest appreciable interaction between FZD3 and Nb1. Thus, the equilibrium dissociation binding constant (K*d)* was not calculated. **b** Comparison of BLI-based binding response for Nb1 (bottom 4 lines) with Nb9 (top 4 lines) at the same corresponding nanobody concentrations.

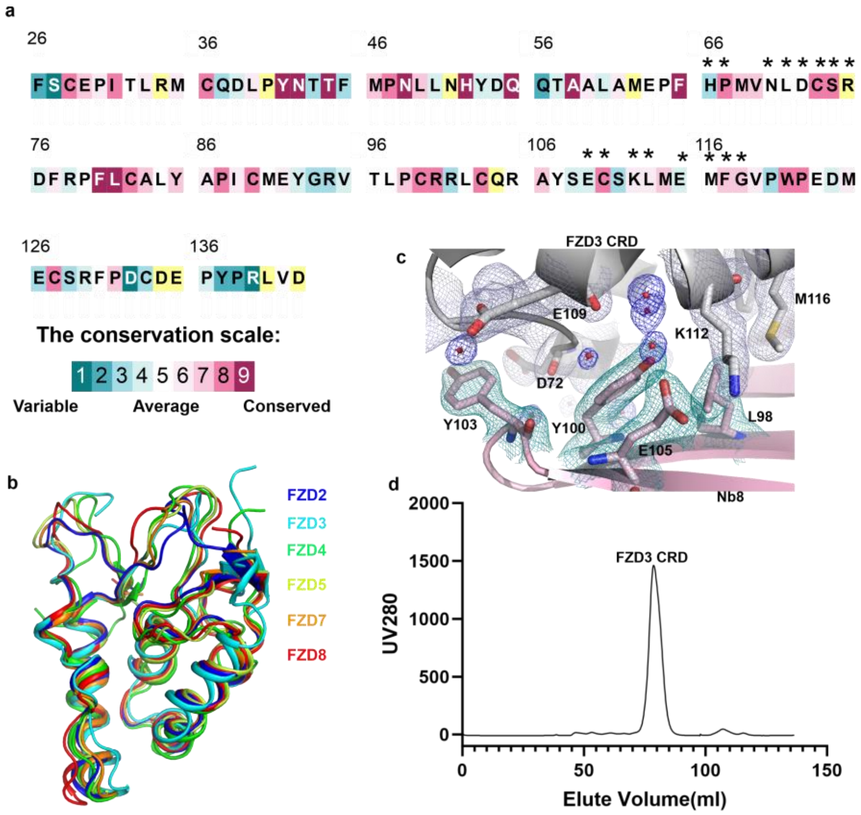

Supplementary Fig. 4: Evolutionary conservation of human FZD CRDs, FZD3 CRD-Nb8 interface and FZD3 CRD protein purification.

a Evolutionary conservation of all ten human FZD CRDs mapped onto the amino acid sequence of FZD3 CRD. FZD3 CRD sequence is coloured based on the conservation level compared to the other 9 human FZD CRDs using ConSurf webserver. FZD3 residues that interact with Nb8 are indicated with asterisks above the sequence. Residues with yellow background indicate that the calculation for this site was performed on less than 10% of the sequences (insufficient data). **b** FZD3 CRD was superimposed with CRDs from FZDs 2, 4, 5, 7, 8 (PDB IDs: 6C0B, 5BPB, 5URY, 5T44, 6TFM) with a root-mean-square deviation (RMSD) of 1.4, 1.6, 1.5, 1.2, 1.4 Å for 113 aligned Cα atoms, respectively. **c** |2Fo – Fc| electron density map for FZD3 CRD (grey cartoon and sticks with light-blue mesh)-Nb8 (pink with teal mesh) interface, contoured at 1.0σ. Waters are shown as red ball with blue mesh. **d** FZD3 CRD protein purification size exclusion chromatography trace. HiLoad 16/600 Superdex 75 pg column was used, and the gel filtration buffer was 10 mM Hepes, pH7.4, 150 mM NaCl.

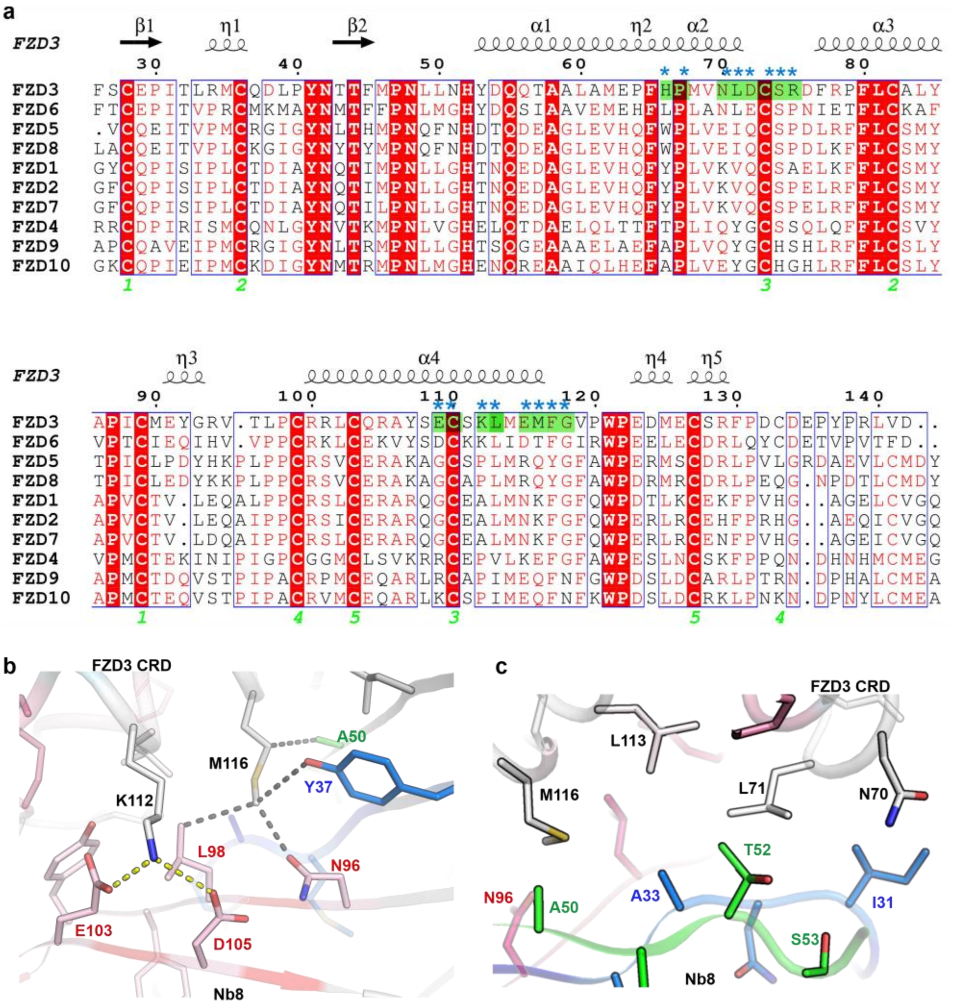

Supplementary Fig. 5: Amino acid sequence alignment of all 10 human FZD CRDs, and zoom-in views of the FZD3 CRD-Nb8 interactions.

a amino acid sequence alignment of all 10 human FZD CRDs. Residue numbering and secondary structure annotation correspond to human FZD3 (UniProt ID Q9NPG1). The conserved residues are highlighted in red. The amino acids responsible for interaction with Nb8 are highlighted in green and marked with asterisks above the sequence. Disulphide bond pairs are indicated by green numbers. The FZD3 amino acids N70, K112 are uniquely shared the FZD6, while the D72, E109, and E115 are substituted with similar residues in the FZD6, these positions are potential epitopes for cross-reactivity of Nb8 with the FZD6. **b** and **c** cartoon representation of FZD3 CRD-Nb8 interactions. The sidechains of interacting residues are shown as sticks. The colour scheme is the same as in Fig. 2. The yellow dashed lines indicate electrostatic interactions (hydrogen bonds), and black dashed lines denote van der Waals (hydrophobic) interactions.

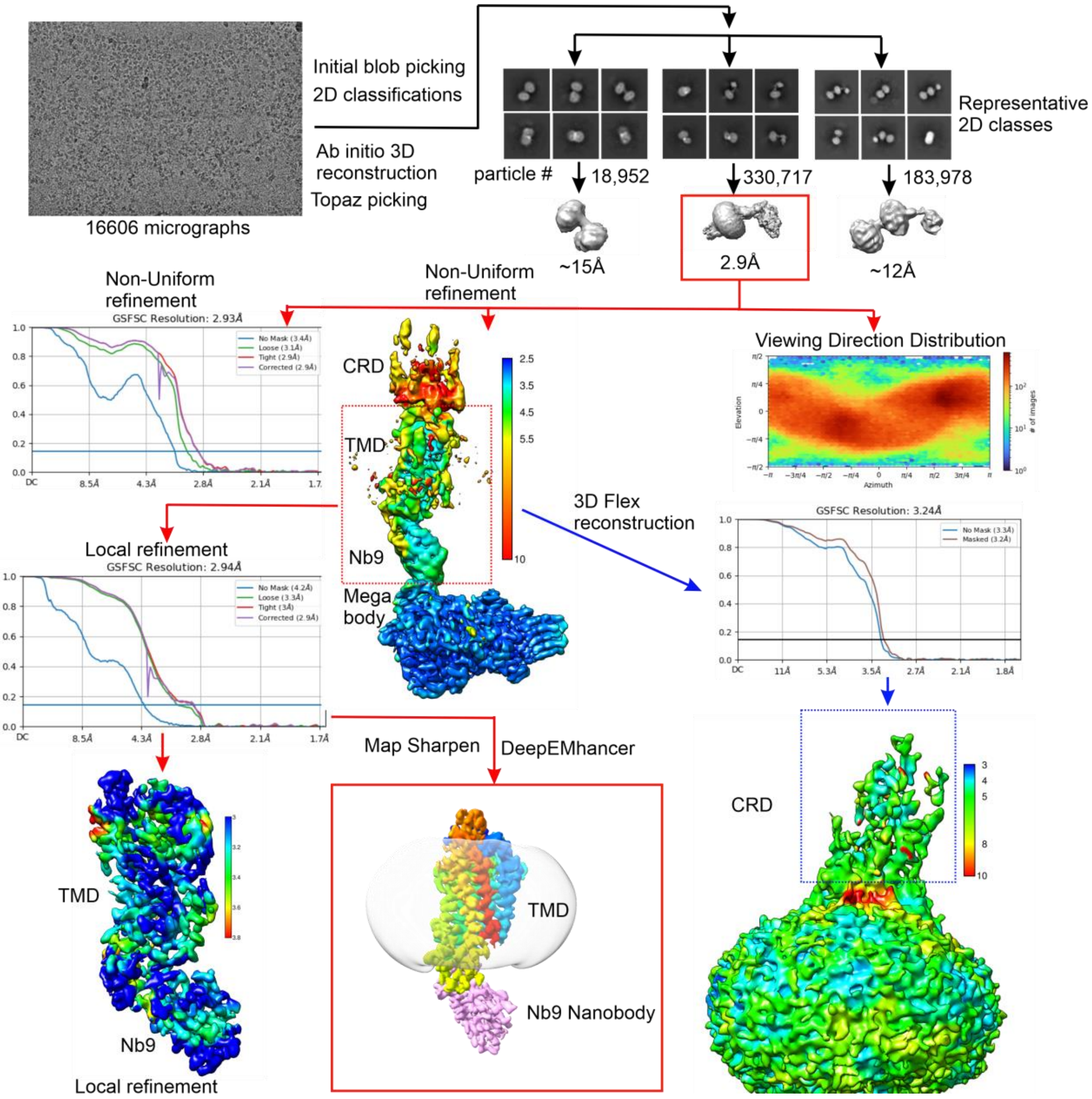

Supplementary Fig. 6: Cryo-EM images and data processing of FZD3-Nb9 megabody complex. A representative cryo-EM micrograph and 2D classes are shown. The 3D density maps are coloured according to their local resolution estimation (Å). The agular distribution of particles is shown. The corresponding Fourier shell correlation curves (FSC, resolution cut-off at 0.143) are shown next to the maps. The final sharpened map (by DeepEMhancer) is shown within the red box with the TM domain rainbow-coloured and Nb9 coloured pink.

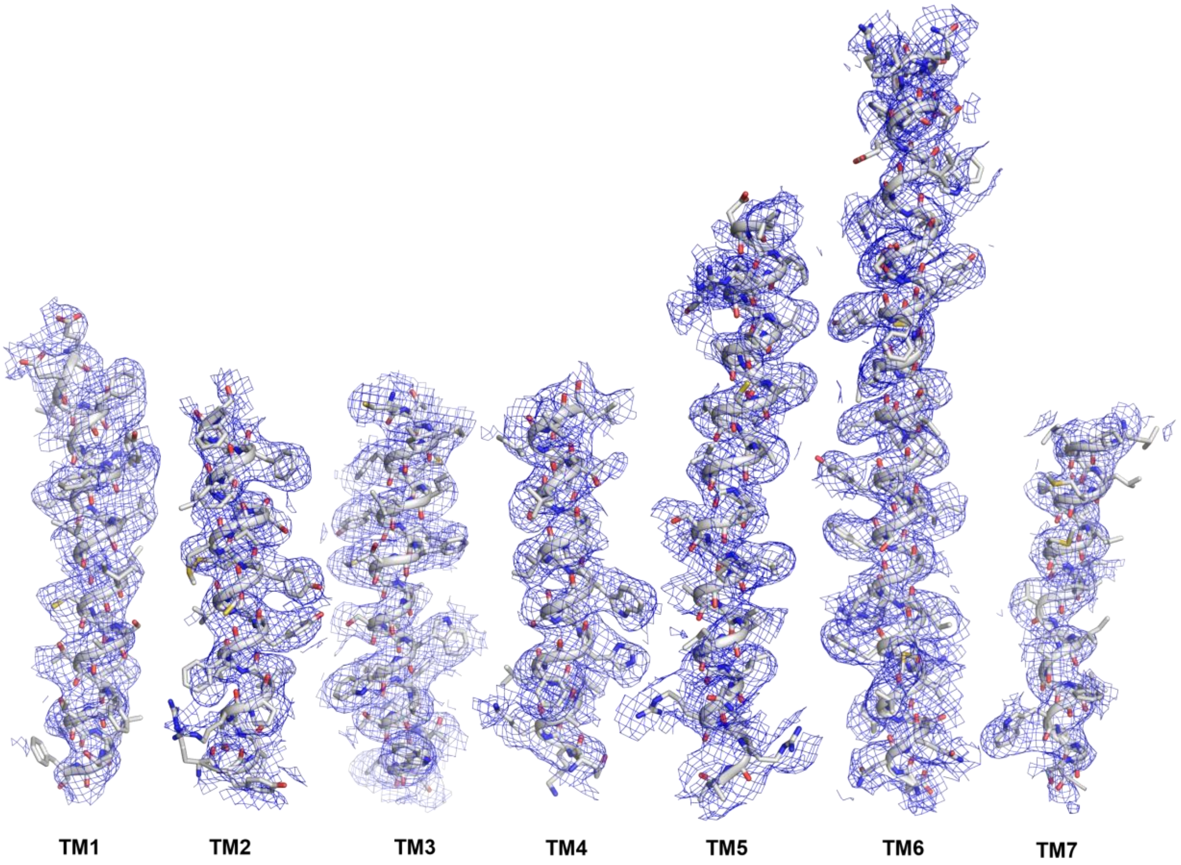

Supplementary Fig. 7: The segmented electron density maps of the trans-membrane helices of FZD3. Side chains of residues are shown as sticks with carbon atoms coloured in grey, nitrogen in blue, oxygen in red, and sulphur in yellow.

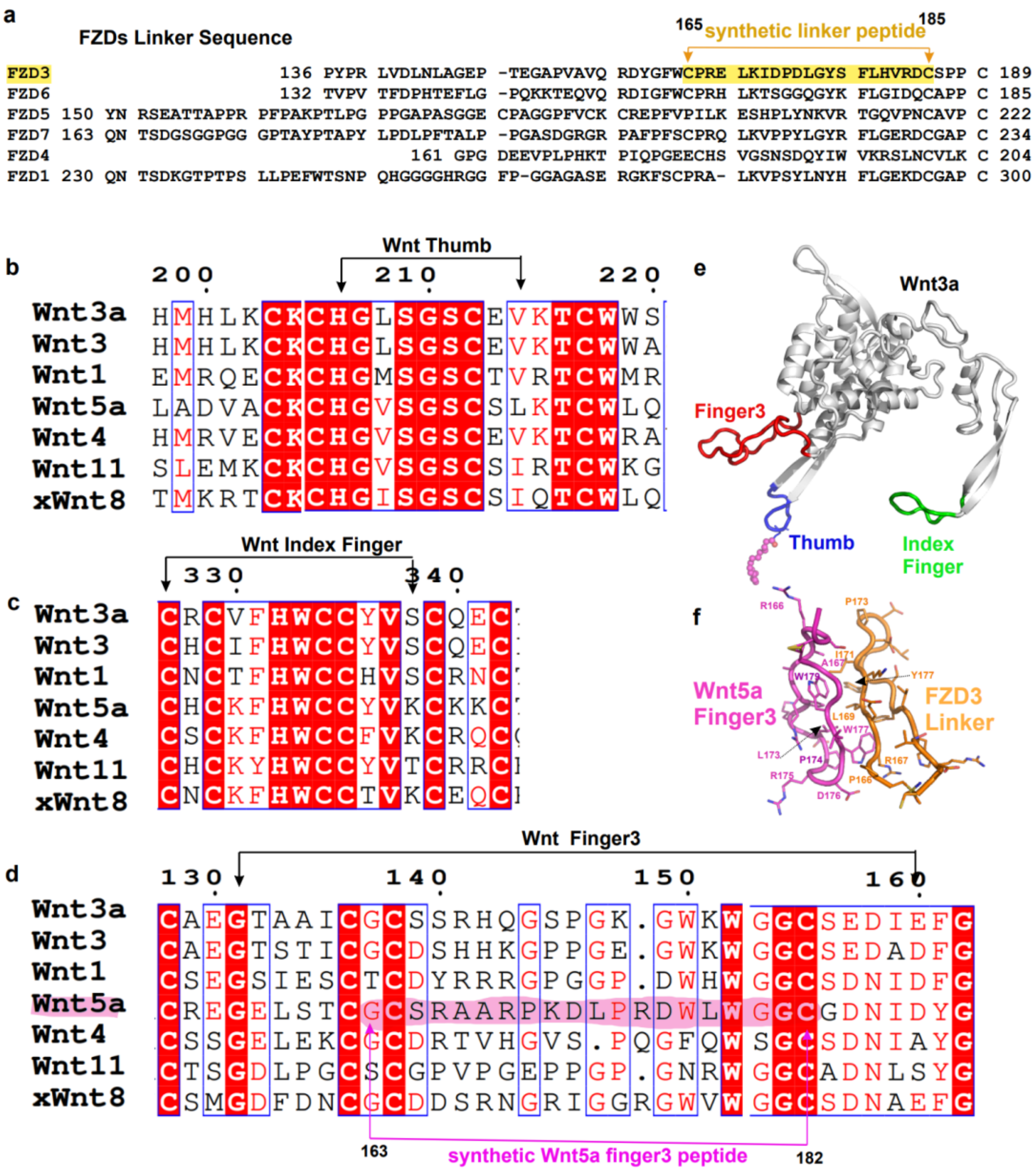

112

113 **Supplementary Fig. 8: Amino acid sequence alignments of FZD linkers and Wnt fingers, as well as cartoon representation of Wnt3a fingers and modelling of Wnt5a-FZD3 interactions**. **a** selected FZD linker sequences, with residue numbers indicated. **b-d** alignment of selected Wnt finger sequences. The residue numbering corresponds to human Wnt3a. Residue numbers of the FZD3 synthetic linker peptide (165-185) and Wnt5a synthetic finger-3 peptide (163-182) are indicated in panels **a** and **d** respectively. **e** cartoon representation of Wnt3a fingers. The magenta spheres denote PAM lipid. **f** AlphaFold model of Wnt5a finger3 in complex with the linker domain of FZD3. The sequences used for AlphaFold-based model prediction are highlighted in panels **a** and **d**.

123

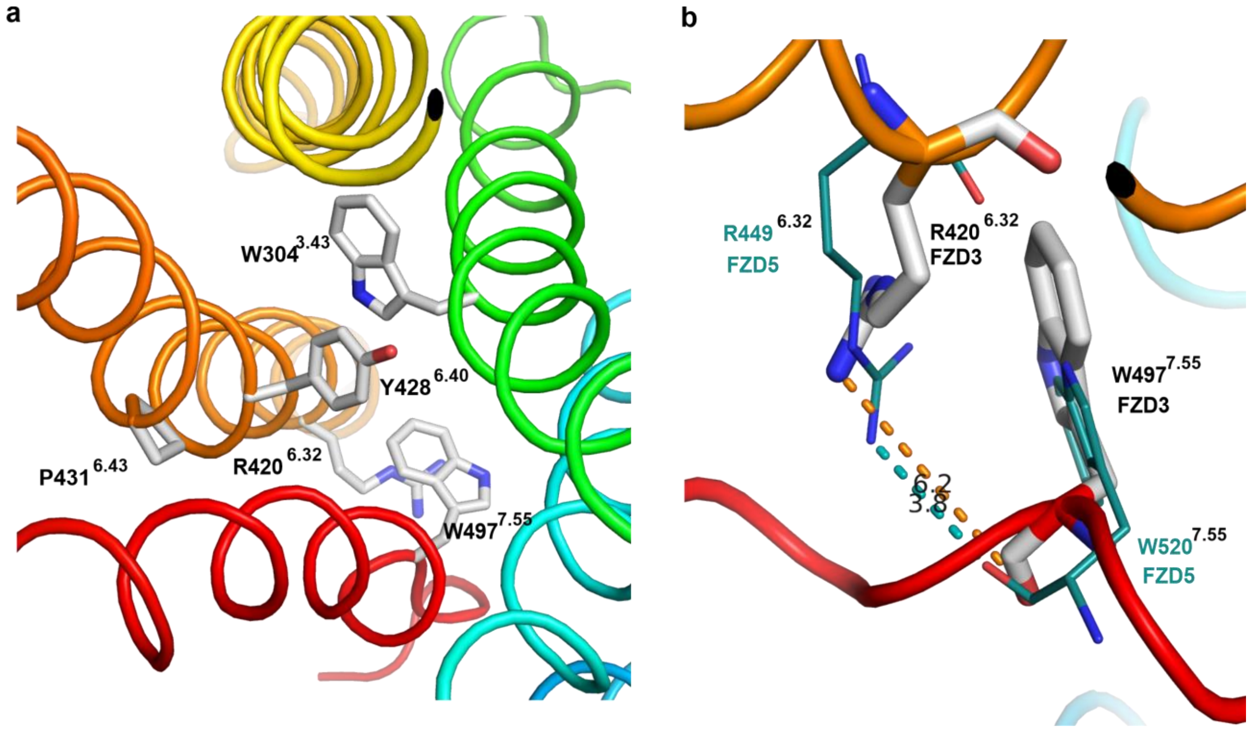

Supplementary Fig. 9: Conserved molecular switch of the FZD3 and comparison with the FZD5

a FZD3 cartoon shows molecular switch residues. **b** alignment of FZD3 and FZD5 (PDB 6WW2, only the relevant residues are shown as thin sticks in teal) showing molecular switch residues R6.32 and W7.55 and distances. Teal dashed line indicates the distance (3.8 Å) in FZD5, and orange dash line indicates the corresponding distance (6.2 Å) in FZD3.

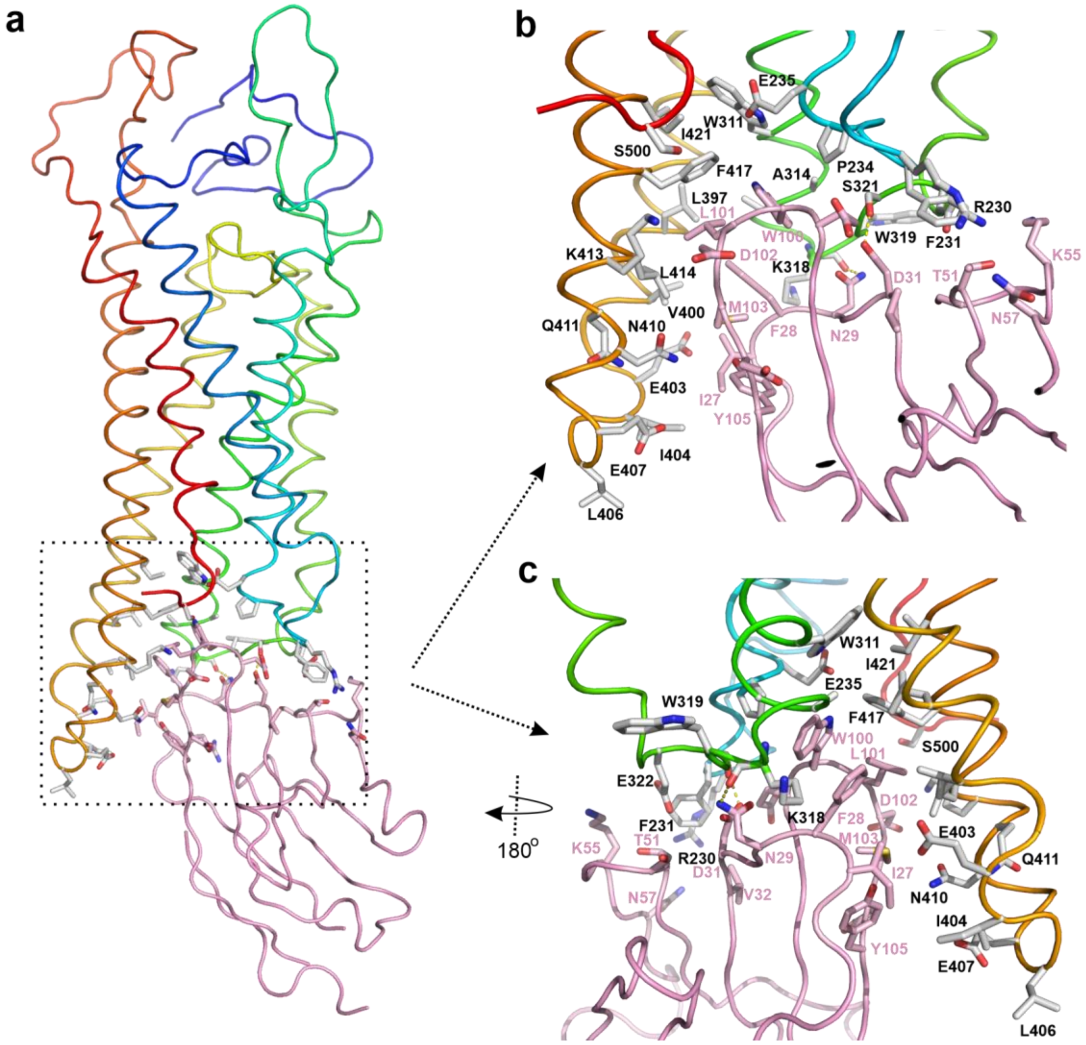

Supplementary Fig. 10: Details of FZD3-Nb9 interactions. **a** FZD3 Trans-membrane domain of FZD3 is shown as rainbow cartoon. Nb9 is shown as pink tube. Side chains of interacting residues are shown as sticks. **b** close-up view indicated in panel **a**. **c** the same close-up region as shown in panel **b** but rotated around y axis by 180°.

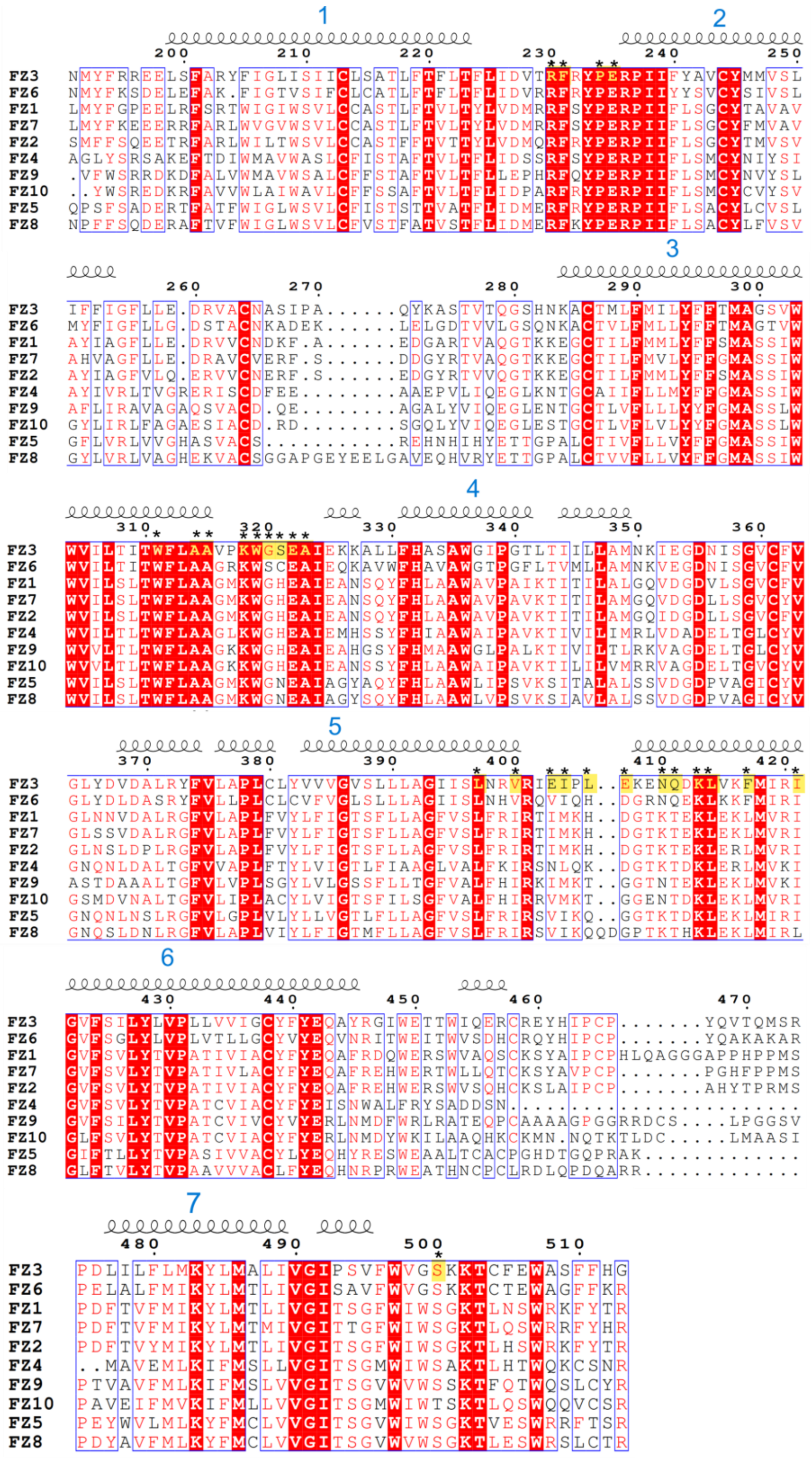

Supplementary Fig. 11: Amino acid sequence alignment of all 10 human FZD transmembrane domains. Residue numbering and secondary structure annotation is based on human FZD3. The conserved residues are coloured in red. The amino acids that interact with Nb9 are highlighted yellow with stars on the top.

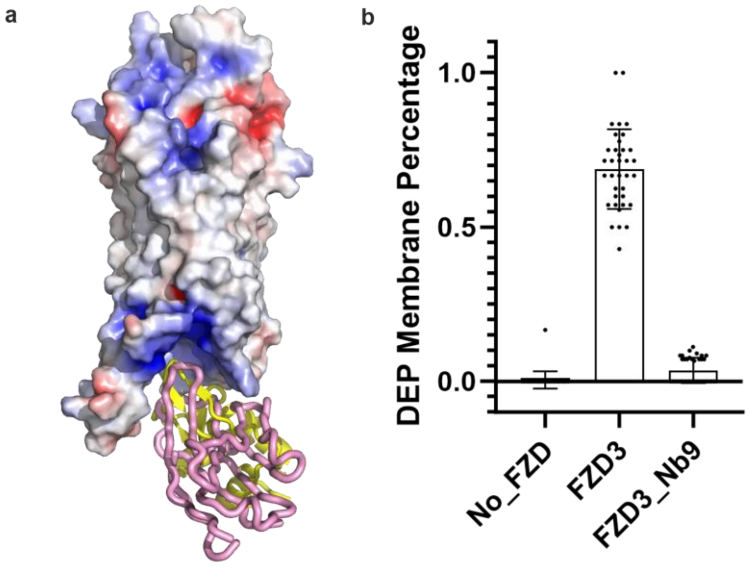

Supplementary Fig. 12: Nb9 overlaps with predicted DEP domain and competes DEP recruit to FZD3.

a The cryo-EM structure of FZD3-Nb9 was superimposed with the AlphaFold predicted complex of FZD3-DEP domain. The TMD is shown as electrostatic surface. The Nb9 is shown as a pink tube. The DEP domain is shown as yellow cartoon. **b** The percentage of cells showing membrane localization of DEP-GFP in HEK293T FZD1-10 knock-out cells (No_FZD), FZD3 transfected cells (FZD3) and FZD3 with C-terminal Nb9 fusion (FZD3_Nb9). Three independent transfections, 12 frames were taken for each sample (n=36). The total cell numbers were 238, 250, 334 for No_FZD, FZD3 and FZD3_Nb9 respectively. Two-tailed t test $p<0.0001$ between FZD3 and FZD3_Nb9. The error bars are presented as mean +/-SD.

Supplementary Table 1: FZD3-CRD Nb8 crystal Data collection and refinement statistics

	FZD3 CRD-Nb8 (PDB 8Q7O)
**Data collection**	
Space group	$C222_1$
Cell dimensions	
a, *b*, *c* (Å)	53.95 146.43 229.51
α, β, γ (°)	90, 90, 90
Resolution (Å)	114.76–1.77 (1.99–1.77)*
R_{sym} or R_{merge}	0.078 (>1)
$I / \sigma I$	18.0 (1.8)
Completeness (%)	93.9 (81.3)
Redundancy	13.8 (14.6)
**Refinement**	
Resolution (Å)	114.76–1.77
No. reflections	663243 (35278)
R_{work} / R_{free}	0.1948/0.2184
No. atoms	4276
Protein	3817
Ligand/ion	129
Water	330
B-factors	
Protein	40.79
Ligand/ion	86.71
Water	43.54
R.m.s. deviations	
Bond lengths (Å)	0.006
Bond angles (°)	1.23

*Values in parentheses are for highest-resolution shell. One crystal was used for this data.

Supplementary Table 2: Cryo-EM data collection refinement and validation statistics

	FZD3-Nb9<br>(EMDB-18680)<br>(PDB 8QW4)
**Data collection and processing**	
Magnification	105
Voltage (kV)	300
Electron exposure (e–/Å^2)	40
Defocus range (μm)	-1 to -2.2
Pixel size (Å)	0.831
Symmetry imposed	C1
Initial particle images (no.)	533467
Final particle images (no.)	330717
FSC threshold	0.143
Map resolution range (Å)	2.5-10
**Refinement**	
Initial model used (PDB code)	7EVW
Model resolution range (Å)	3-3.8
FSC threshold	0.143
Map sharpening *B* factor (Å^2)	n/a(DeepEMhancer)
Non-hydrogen atoms	3481
Protein residues	434
Ligands	0
B factors (Å2)	
Protein	66.42
Ligand	n/a
R.m.s. deviations	
Bond lengths (Å)	0.007
Bond angles (°)	1.111
**Validation**	
MolProbity score	
Clashscore	11.73
Poor rotamers (%)	0.26
Ramachandran plot	
Favored (%)	85.95
Allowed (%)	14.05
Disallowed (%)	0
