# Supplementary material for: Structural insights into Frizzled3 through nanobody modulators
Source: Nat Commun. 2024 Aug 22;15:7228. doi: 10.1038/s41467-024-51451-1 (PMC11341715; doi:10.1038/s41467-024-51451-1)
Supplement: Supplementary file 2 — Reporting Summary [file 41467_2024_51451_MOESM2_ESM.pdf]

nature portfolio

Corresponding author(s): E. Yvonne Jones

Last updated by author(s): 25th July, 2024

Reporting Summary

Nature Portfolio wishes to improve the reproducibility of the work that we publish. This form provides structure for consistency and transparency in reporting. For further information on Nature Portfolio policies, see our Editorial Policies and the Editorial Policy Checklist.

Statistics

For all statistical analyses, confirm that the following items are present in the figure legend, table legend, main text, or Methods section.

n/a	Confirmed	
[ ]	[x]	The exact sample size (*n*) for each experimental group/condition, given as a discrete number and unit of measurement
[ ]	[x]	A statement on whether measurements were taken from distinct samples or whether the same sample was measured repeatedly
[ ]	[x]	The statistical test(s) used AND whether they are one- or two-sided<br>*Only common tests should be described solely by name; describe more complex techniques in the Methods section.*
[x]	[ ]	A description of all covariates tested
[x]	[ ]	A description of any assumptions or corrections, such as tests of normality and adjustment for multiple comparisons
[ ]	[x]	A full description of the statistical parameters including central tendency (e.g. means) or other basic estimates (e.g. regression coefficient) AND variation (e.g. standard deviation) or associated estimates of uncertainty (e.g. confidence intervals)
[ ]	[x]	For null hypothesis testing, the test statistic (e.g. *F*, *t*, *r*) with confidence intervals, effect sizes, degrees of freedom and *P* value noted<br>*Give P values as exact values whenever suitable.*
[x]	[ ]	For Bayesian analysis, information on the choice of priors and Markov chain Monte Carlo settings
[x]	[ ]	For hierarchical and complex designs, identification of the appropriate level for tests and full reporting of outcomes
[x]	[ ]	Estimates of effect sizes (e.g. Cohen's *d*, Pearson's *r*), indicating how they were calculated

Our web collection on statistics for biologists contains articles on many of the points above.

Software and code

Policy information about availability of computer code

Data collection	For X-ray crystal data collection, standard software GDA (Generic Data Acquisition) was used.<br>For Cryo EM data collection, standard EPU (version 3.1.0) was used. No additional commercial or custom code used.
Data analysis	For X-ray crystal data analysis, Phenix (Python-based Hierarchical ENvironment for Integrated Xtallography) version 1.20.1 was used.<br>For Cryo EM data analysis, standard cryoSPARC, version 4.4.1 was used.

For manuscripts utilizing custom algorithms or software that are central to the research but not yet described in published literature, software must be made available to editors and reviewers. We strongly encourage code deposition in a community repository (e.g. GitHub). See the Nature Portfolio guidelines for submitting code & software for further information.

Data

Policy information about availability of data

All manuscripts must include a data availability statement. This statement should provide the following information, where applicable:

- Accession codes, unique identifiers, or web links for publicly available datasets
- A description of any restrictions on data availability
- For clinical datasets or third party data, please ensure that the statement adheres to our policy

The coordinates have been deposited in the Protein Data Bank (PDB) under accession codes 8Q7O for the FZD3 CRD-Nb8 complex and 8QW4 for the FZD3-Nb9

complex. The cryo-EM maps have been deposited in the Electron Microscopy Data Bank (EMDB) under accession code EMD-18680.

Research involving human participants, their data, or biological material

Policy information about studies with human participants or human data. See also policy information about sex, gender (identity/presentation), and sexual orientation and race, ethnicity and racism.

Reporting on sex and gender	N/A
Reporting on race, ethnicity, or other socially relevant groupings	N/A
Population characteristics	N/A
Recruitment	N/A
Ethics oversight	N/A

Note that full information on the approval of the study protocol must also be provided in the manuscript.

Field-specific reporting

Please select the one below that is the best fit for your research. If you are not sure, read the appropriate sections before making your selection.

☒ Life sciences ☐ Behavioural & social sciences ☐ Ecological, evolutionary & environmental sciences

For a reference copy of the document with all sections, see nature.com/documents/nr-reporting-summary-flat.pdf

Life sciences study design

All studies must disclose on these points even when the disclosure is negative.

Sample size	For Fig.2f TOP FLASH luciferase assay, n=9, from 3 independent experiments of triplicates. This size is very much a standard size for this well known assay.<br>For Fig5c BRET assay, n=12, from 3 independent experiments of quadruplicates. For this assay, standard size is 3, we increase 1.<br>For Fig4 h-j related Supplementary Fig12d, DEP image analysis, n=36, 3 independent experiments of 12 frames (total cell numbers were 238, 250 and 334 respectively).Data is presented as per frame counts.
Data exclusions	None
Replication	For Fig.2f TOP FLASH assay, the cell status, seeding concentration, transfection procedure and time points for the assays are tightly kept to ensure good replication. All attempts at replication were successful.<br>For Fig5c BRET assay, the assay itself is very well internal controlled, not influenced by transfection variations. We make sure cells are in good status, and all attempts at replication were successful.<br>For Fig4 h-j related Supplementary Fig12d, DEP image analysis, only the transfected florescent cells were take into account, so less variations from the transfection rate. We make sure cells are in good status. All attempts at replication were successful.
Randomization	It is not relevant to the assays here.
Blinding	It is not relevant to the assays here.

Reporting for specific materials, systems and methods

We require information from authors about some types of materials, experimental systems and methods used in many studies. Here, indicate whether each material, system or method listed is relevant to your study. If you are not sure if a list item applies to your research, read the appropriate section before selecting a response.

Materials & experimental systems

n/a	Involved in the study
[x]	[ ] Antibodies
[ ]	[x] Eukaryotic cell lines
[x]	[ ] Palaeontology and archaeology
[x]	[ ] Animals and other organisms
[x]	[ ] Clinical data
[x]	[ ] Dual use research of concern
[x]	[ ] Plants

Methods

n/a	Involved in the study
[x]	[ ] ChIP-seq
[x]	[ ] Flow cytometry
[x]	[ ] MRI-based neuroimaging

Eukaryotic cell lines

Policy information about cell lines and Sex and Gender in Research

Cell line source(s)	ATCC (American Type Culture Collection)
Authentication	None
Mycoplasma contamination	Tested negative
Commonly misidentified lines (See ICLAC register)	None

Plants

Seed stocks	N/A
Novel plant genotypes	N/A
Authentication	N/A
